# The ability of whole-body SUVmax in F-18 FDG PET/CT to predict suboptimal cytoreduction during primary debulking surgery for advanced ovarian cancer

**DOI:** 10.1186/s13048-019-0488-2

**Published:** 2019-02-04

**Authors:** Gun Oh. Chong, Shin Young Jeong, Yoon Hee Lee, Hyun Jung Lee, Sang-Woo Lee, Hyung Soo Han, Dae Gy Hong, Yoon Soon Lee

**Affiliations:** 1 Department of Obstetrics and Gynecology, School of Medicine, Kyungpook National University, Daegu, 41944 Republic of Korea; 2 Department of Nuclear Medicine, School of Medicine, Kyungpook National University, 130 Dongdeok-ro, Jung-gu, Daegu, 41944 Republic of Korea; 3 Department of Physiology, School of Medicine, Kyungpook National University, Daegu, 41944 Republic of Korea; 4 Molecular Diagnostics and Imaging Center, School of Medicine, Kyungpook National University, Daegu, 41944 Republic of Korea; 5 Department of Obstetrics and Gynecology, Kyungpook National University Chilgok Hospital, Daegu, 41404 Republic of Korea; 6 Department of Nuclear Medicine, Kyungpook National University Chilgok Hospital, Daegu, 41404 Republic of Korea

**Keywords:** Advanced ovarian cancer, Suboptimal cytoreduction, SUVmax, FDG PET/CT, Risk model

## Abstract

**Background:**

The aim of this study was to (1) evaluate the ability of F-18 fluorodeoxyglucose positron emission tomography/computed tomography (FDG PET/CT) parameters to predict suboptimal cytoreduction and (2) to create a risk model for predicting suboptimal cytoreduction in advanced ovarian cancer. From 2011 to 2015, 51 patients underwent primary cytoreductive surgery for advanced ovarian cancer were enrolled. A residual disease with maximal diameter >  1 cm was considered a suboptimal surgical result. The SUVmax values for nine abdominal regions, the sum of 9 regional SUVmax (WB1SUVmax) and WB2SUVmax (WB1SUVmax plus SUVmax of lymph nodes) were used for PET parameter. Multiple logistic regression analysis was used to determine the predictive value of PET and clinical parameters for risk model. In addition, assessments of disease-free survival (DFS) and overall survival (OS) were performed.

**Results:**

Seventeen of the 51 patients (33.3%) underwent suboptimal cytoreduction. The ECOG status (OR, 4.091), SUVmax of central (OR, 5.250), right upper (OR, 4.148), left upper (OR, 5.921) and WB2SUVmax (OR, 4.148) were associated with suboptimal cytoreduction. The risk model can divide the risk groups of suboptimal cytoreduction (area under the curve (AUC), 0.775; *p* = 0.0001). The DFS and OS in the high-risk group were significantly worse than those in the low-risk group (*p* = 0.0379 for DFS; *p* = 0.0211 for OS).

**Conclusions:**

The presence of hypermetabolic lesions in the central, right upper, and left upper regions showed predictive value for suboptimal cytoreduction. Our risk model may be helpful for selecting patients who may show suboptimal cytoreduction.

## Background

Ovarian cancer is one of the most lethal gynecologic cancers [1]. More than 70% of patients with ovarian cancer are diagnosed at an advanced stage (International Federation of Gynecology and Obstetrics [FIGO] stage III or IV), and the 5-year survival rate is less than 30% [2]. To improve the survival of advanced ovarian cancer patients, optimal cytoreductive surgery (e.g., maximum diameter of 1 cm or less for the largest residual tumor mass) followed by postoperative platinum/taxane combination chemotherapy are the cornerstones of treatment [3, 4]. The success rate for achievement of optimal cytoreduction varies widely, from 15 to 85%, in the literature [5]. However, if patients fail to achieve optimal cytoreduction, they may experience major morbidity without any survival benefit [6]. Therefore, a significant proportion of women with advanced ovarian cancer may undergo a debulking operation and experience the associated morbidity without any survival gains. Neoadjuvant chemotherapy followed by interval debulking surgery is an alternative treatment option to overcome situations in which it is difficult to achieve primary optimal cytoreduction [7]. Therefore, precise pretreatment discrimination of patients who are not amenable to optimal cytoreduction may be one of the principal determinants to reduce surgical morbidity.

In order to identify the patients who are less likely to benefit from primary cytoreductive surgery, several attempts have been made to determine specific predictors of cytoreductive outcome, including imaging modalities, tumor markers, and laparoscopic scores [8–10]. F-18 fluorodeoxyglucose positron emission tomography/computed tomography (FDG PET/CT) may be helpful in validating the selection and management of recurrent ovarian cancer patients by identification of extra-abdominal disease and may allow adequate planning of surgical debulking at retroperitoneal, hepatic, or splenic sites [11, 12]. However, the role of FDG PET/CT in predicting the outcome of primary optimal cytoreductive surgery for advanced ovarian cancer has not been reported.

The maximum standardized uptake (SUVmax) value reflects the degree of glucose metabolism within the tumor, which represents the aggressiveness of the malignant lesion. Therefore, we hypothesized that the sum of the SUVmax values for the whole abdominal pelvic cavity would better represent tumor activity in advanced ovarian cancer. To test this hypothesis, we obtained the SUVmax for nine different regions in the abdominal pelvic cavity. The aim of this study was to evaluate the predictive value of SUVmax values on FDG PET/CT for suboptimal cytoreduction in advanced ovarian cancer and to create a risk model using metabolic parameters for predicting suboptimal cytoreduction.

## Results

### Clinical features and surgical outcomes

The patients’ median age at diagnosis was 58 years (range, 37–74 years). Thirty-seven patients (72.5%) presented with FIGO stage III ovarian cancer and 14 patients (27.5%) presented with stage IV disease. Grade 3 lesions were found in 82.4% of the patients. The predominant tumor histology was serous differentiation (78.4%, Table 1).

**Table 1:** Patient and tumor characteristics

Variable	Median (range) or Number (%)
Age (years)	58.0 (37–74)
FIGO stage	
IIIC	37 (72.5)
IV	14 (27.5)
Tumor grade	
1	1 (2.0)
2	8 (15.7)
3	42 (82.4)
Histology	
Serous	40 (78.4)
Endometroid	5 (9.8)
Clear	2 (3.9)
Mucinous	1 (2.0)
Others	3 (5.9)
Preoperative CA 125 (U/mL)	573.7 (38–6000)
Ascites	
< 500 mL	11 (21.6)
> 500 mL	40 (78.4)
ECOG PS	
0	8 (15.7)
1	31 (60.8)
2	12 (23.6)

*FIGO* International Federation of Gynecology and Obstetrics, *ECOG PS* Eastern cooperative oncology group performance status

Optimal cytoreduction was achieved in 34 patients (66.7%); however; 17 patients (33.3%) had > 1-cm residual disease. The median operating time was 400 min (range, 170–720 min). The detailed surgical procedures for optimal cytoreductive surgery are listed in Table 2. The main postoperative complications were infection (27.5%), followed by wound disruption (19.6%), intestinal complications such as ileus or anastomotic insufficiency, and thromboembolism (5.9%) (Table 2).

**Table 2:** Surgical outcomes and procedures to achieve optimal cytoreduction

Variables	Median (range) or Number (%)
Operating time (minutes)	400.0 (120–720)
Optimal cytoreduction	34 (66.7)
Suboptimal cytoreduction	17 (33.3)
Surgical procedures	
Hysterectomy	49 (96.1)
Adenectomy	51 (100.0)
Omentectomy	51 (100.0)
Peritonectomy	39 (76.5)
Pelvic Lymphadenectomy	42 (82.4)
Paraaortic Lymphadenectomy	42 (82.4)
Small bowel resection	4 (7.8)
Large bowel resection	23 (45.1)
Ileostomy	10 (19.6)
Appendectomy	38 (74.5)
Partial liver resection	9 (17.6)
Cholecystectomy	14 (27.5)
Splenectomy	14 (27.5)
Diaphragm resection	33 (64.7)
Partial bladder resection	2 (3.9)
Postoperative complication	
Intestinal complications (Ileus, anastomotic insufficiency)	5 (9.8)
Infection	14 (27.5)
Wound disruption	10 (19.6)
Thromboembolism	3 (5.9)

### Cut-off values for metabolic parameters

The optimal cut-off values for PET 0 – PET 8, WB1SUVmax, and WB2SUVmax were calculated using the Receiver operating characteristic (ROC) curve. The optimal cut-off values for each PET parameter according to regions were 2.2 for PET0, 7.9 for PET 1, 5.3 for PET 2, 0 for PET 3, 1.8 for PET 4, 4.3 for PET 5, 6.7 for PET 6, 4.5 for PET 7, and 6 for PET 8. The optimal cut-off WB1SUVmax value was 68.8 and that for WB2SUVmax was 76.2 (Table 3).

**Table 3:** Cut-off values and univariate analysis of PET metabolic parameters for prediction of suboptimal cytoreduction

	Median (range)	Cut-off value	AUC	*P* value	OR	95% CI	*P* value	Predictive score
PET 0	3.5 (0.0–16.0)	> 2.2	0.670	0.0277	5.250	1.41–19.59	0.0136	5
PET 1	4.0 (0.0–14.3)	> 7.9	0.657	0.0563	4.148	1.13–15.19	0.0317	4
PET 2	4.5 (0.0–23.0)	> 5.3	0.529	0.7387	0.440	0.12–1.63	0.2196	
PET 3	3.8 (0.0–18.2)	> 0	0.652	0.0546	5.921	1.17–30.02	0.0318	6
PET 4	2.9 (0.0–19.0)	> 1.8	0.593	0.2599	2.889	0.78–10.68	0.1118	
PET 5	4.9 (0.0–18.0)	> 4.3	0.500	1.000	0.553	0.17–1.80	0.3244	
PET 6	10.0 (0.0–24.2)	> 6.7	0.510	0.9097	0.393	0.10–1.48	0.1683	
PET 7	6.1 (0.0–13.2)	> 4.5	0.506	0.9450	0.425	0.13–1.40	0.1599	
PET 8	5.7 (0.0–16.8)	> 6	0.631	0.1224	1.118	0.97–1.29	0.1180	
WB1SUVmax	46.4 (7.4–134.0)	> 68.8	0.621	0.1644	3.429	0.97–12.14	0.0561	
WB2SUVmax	53.3 (7.4–153.0)	> 76.2	0.621	0.1957	4.148	1.13–15.19	0.0317	4
PCI	22.0 (7.0–32.0)	> 10	0.562	0.4448	5.760	0.66–49.91	0.1120	
Age	58.0 (37–74)	≥ 60	0.500	1.000	1.000	0.30–3.26	1.0000	
Ascites	NA	> 500	0.515	0.8173	0.842	0.21–3.40	0.8107	
Ca 125	573.7 (38–6000)	> 1881	0.568	0.4595	2.133	0.62–7.39	0.2221	
ECOG PS	NA	≥2	0.618	0.0746	4.091	0.97–17.29	0.0520	4
Stage	NA	IV	0.603	0.1464	2.70	0.75–9.66	0.1265	

*PET* Positron emission tomography, *WB1SUVmax* Whole-body 1 standardized uptake value, *WB2SUVmax* Whole-body 2 standardized uptake value, *PCI* Peritoneal cancer index, *ECOG PS* Eastern cooperative oncology group performance status, *NA* Not available, *AUC* Area under the curve, *OR* Odds ratio, *95% CI* 95% confidence interval

### Correlation between suboptimal cytoreduction and clinical and PET metabolic parameters

According to the univariate analysis, among the clinical parameters, only ECOG status was associated with suboptimal cytoreduction with marginal significance (odds ratio (OR), 4.091; 95% CI, 0.97–17.29; *p* = 0.0520). Among the PET metabolic parameters, PET 0 (central: OR, 5.250; 95% CI, 1.41–19.59; *p* = 0.0136), PET 1 (right upper: OR, 4.148; 95% CI, 1.13–15.19; *p* = 0.0317), and PET 3 (left upper: OR, 5.921; 95% CI, 1.17–30.02; *p* = 0.0318) were significantly associated with suboptimal cytoreduction. Moreover, WB2SUVmax was a significant predictive factor for suboptimal cytoreduction (OR, 4.148; 95% CI, 1.13–15.19, *p* = 0.0317). However, no correlation was found between peritoneal cancer index (PCI) and suboptimal cytoreduction (OR, 5.760; 95% CI, 0.66–49.94; *p* = 0.1120) (Table 3).

### Prediction model for suboptimal cytoreduction and survival analyses

The clinical and PET metabolic parameters found to be significant in univariate analysis were then each assigned a “predictive score” according to their OR. The predictive scores were as follows: PET 0 = 5, PET 1 = 4, PET 3 = 6, WB2SUVmax = 4, and ECOG status = 4.

**Fig. 1:**
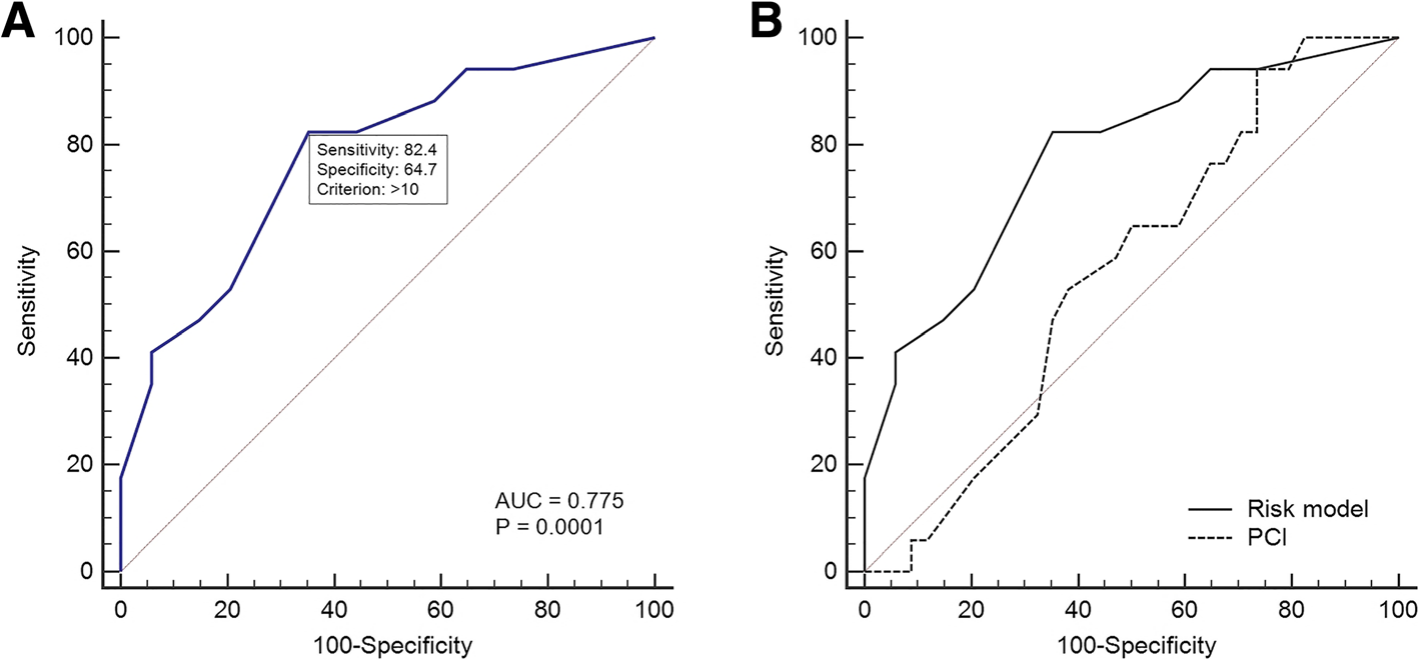
**a**. Receiver operating characteristic curve analysis of the risk model for predicting suboptimal cytoreduction. The area under the curve was 0.775 (95% confidence interval, 0.64–0.88; *p* = 0.0001). **b**. Comparison of the receiver operating characteristic curve findings between our risk model and the conventional peritoneal cancer index. The area under the curve was 0.775 for the risk model and 0.562 for the peritoneal cancer index (*p* = 0.0311)

**Fig. 2:**
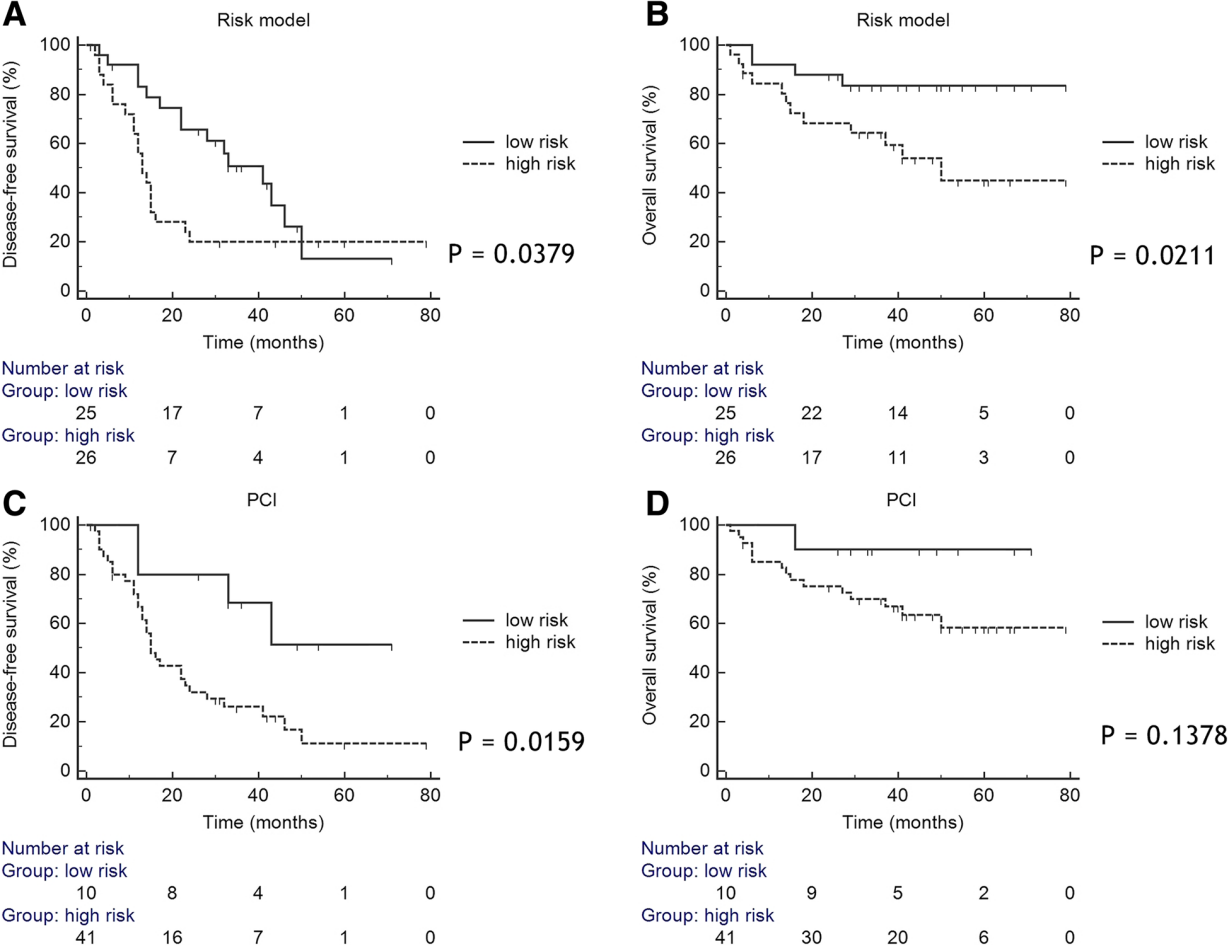
Kaplan-Meier survival plots of disease-free survival and overall survival according to the risk group in the risk model (**a**, **b**) and peritoneal cancer index (**c**, **d**)

According to the ROC curve analysis for the prediction of suboptimal cytoreduction as the sum of each predictive score, 10 was determined to be the cut-off value. The AUC was 0.775 (95% CI, 0.64–0.88; *p* = 0.0001). Sensitivity was 82.4% and specificity was 64.7% (Fig. 1a). A high-risk group for suboptimal cytoreduction was defined by a predictive score >  10, and a low-risk group was defined by a predictive score ≤ 10. Kaplan-Meier survival plots showed that the DFS and OS of the high-risk group were significantly worse than those of the low-risk group (*p* = 0.0379 for DFS; *p* = 0.0211 for OS) (Fig. 2a, b). Complication rates were 53.8% in high-risk group and 32.8% in low-risk group; however, there was no statistically difference (*p* = 0.1189).

### Comparison of the predictive ability of conventional PCI and the prediction model using metabolic parameters for prediction of suboptimal cytoreduction and survival outcomes

In the ROC curve analysis for PCI-based prediction of suboptimal cytoreduction, 10 was determined to be the cut-off value. The AUC was 0.562 (95% CI, 0.42–0.70; *p* = 0.4448). Our risk model using metabolic parameters was superior to conventional PCI for predicting suboptimal cytoreduction (AUC: 0.775 vs. 0.562; *p* = 0.0311) (Fig. 1b). The high-risk groups in both the risk model and PCI-based assessments showed worse DFS than the low-risk groups. However, only the risk model showed a significant difference in terms of OS (Fig. 2).

## Discussion

In this study, we developed a predictive model to identify suboptimal cytoreduction in advanced ovarian cancer using metabolic parameters on FDG PET/CT. Our risk model showed better predictive value than conventional PCI-based assessments for suboptimal cytoreduction. Moreover, the high-risk group in the risk model showed worse DFS and OS than the low-risk group.

Previously, several attempts have been made to predict suboptimal cytoreduction to minimize surgical morbidity. The imaging modality most commonly used to predict the outcome of cytoreductive surgery is CT. Several risk models have demonstrated predictive index scores using the tumor burden at each anatomical position [13–15]. Although CT showed relatively high accuracy for prediction of suboptimal cytoreduction, the parameters and anatomical distribution for each risk model were different and complicated. Thus, it may be difficult to standardize the risk model using CT. Laparoscopy-based assessment has recently gained popularity for prediction of suboptimal cytoreduction. Petrillo et al. proposed a predictive index model based on objective parameters determined during pre-cytoreduction laparoscopy [16]. The advantages of laparoscopic evaluation prior to cytoreductive surgery include the fact that this approach could spare patients an unnecessary laparotomy, and the resultant suboptimal cytoreduction, and allow adjustment of immediate neoadjuvant chemotherapy without requiring recovery from laparotomy. Furthermore, laparoscopy allows collection of tissue for definitive diagnosis and for molecular analyses [17]. However, laparoscopic procedures are more invasive than imaging modalities, and a laparoscopy-based predictive index model has a learning curve. Moreover, the predictive results may differ according to the subjective scoring performed by each surgeon.

The role of FDG PET/CT in the management of advanced ovarian cancer is not well established, especially in predicting suboptimal cytoreduction. Most previous studies focused on prediction of optimal debulking in recurrent ovarian cancer [18, 19]. Fogotti et al. assessed the predictive role of FDG PET/CT in guiding optimal cytoreduction in patients with recurrent ovarian cancer and reported an accuracy of 79%. Thus, they concluded that FDG PET/CT had good efficacy in planning surgical treatment in patients with recurrent ovarian cancer [18]. Moreover, a recent study showed that volume-based quantitative FDG PET/CT metrics that reflect the metabolic tumor burden were associated with optimal secondary cytoreduction in patients with recurrent ovarian cancer [19]. However, there have been only a few studies concerning the role of FDG PET/CT in predicting primary suboptimal cytoreduction. Moreover, the concept of WBSUVmax for predicting suboptimal cytoreduction has never been reported. In this study, WBSUVmax was significantly associated with suboptimal cytoreduction. Furthermore, the presence of hypermetabolic lesions in the central (PET 0), right upper (PET 1), and left upper (PET 3) regions showed predictive value for suboptimal cytoreduction. Central lesions include small bowel mesentery or omental disease, right upper lesions include liver or diaphragm lesions, and left upper lesions include spleen, stomach, or lesser sac lesions. Most previous studies using CT included lesions of the mesentery, liver, small bowel, spleen, and/or stomach as predictors of suboptimal cytoreduction [13–15]. These results are consistent with our study.

Recent studies showed that quantitative assessment by metabolic tumor volume (MTV) rather than by SUVmax may be helpful for stratifying patients who present with peritoneal carcinomatosis from ovarian cancer [20]. Moreover, the MTV survival analysis showed a significantly higher OS in patients presenting with a higher tumor burden than in those with less tumor burden (*p* = 0.06) [20]. Quantitative metabolic parameters such as MTV may better represent the total tumor burden and metabolic activity. In cases involving advanced ovarian cancer, however, the tumor burdens are large and the lesions are located at multiple anatomic sites. Therefore, exact measurement of quantitative metabolic parameters is very difficult and time-consuming. We consider that the sum of the SUVmax values for the nine anatomical regions of the abdominal pelvic cavity may better represent tumor activity than the voxel-based assessment of the SUVmax. The measurement of WBSUVmax may be more convenient and easy than assessments of quantitative metabolic parameters. Moreover, our risk model is relatively simple, objective, less time consuming, and quantifiable compared to CT or laparoscopy for predicting suboptimal debulking. So, our risk model may be used screening tool to predict suboptimal debulking. Furthermore, our risk model may be used to select which patients can be applied the minimally invasive surgery such as laparoscopy and robot. To evaluate the low-risk patient who can conduct the minimally invasive surgery, all stage of ovarian cancer should be included and further study may be needed.

Our study has some noteworthy limitations. First, it was a retrospective study with a limited number of patients. Second, there was no comparison between FDG PET parameters and other imaging modalities or laparoscopy.

## Conclusion

WBSUVmax was significantly associated with suboptimal cytoreduction. Furthermore, hypermetabolic lesions in the central, right upper, and left upper regions showed predictive value for suboptimal debulking. Moreover, our risk model may be helpful for selecting patients who may show suboptimal cytoreduction with other imaging modalities and laparoscopic procedures.

## Methods

### Patients

Retrospective data collection and analysis were approved by the Institutional Review Board of Kyungpook National University Chilgok Hospital. The need for informed consent was waived due to the retrospective design of the study. The patients were staged according to the FIGO staging system. Eastern cooperative oncology group performance status (ECOG PS) was defined as follow: Grade 0; Fully active, able to carry on all pre-disease performance without restriction, Grade 1; Restricted in physically strenuous activity but ambulatory and able to carry out work of a light or sedentary nature, Grade 2; Ambulatory and capable of all self-care but unable to carry out any work activities. Up and about more than 50% of waking hours, Grade 3; Capable of only limited self-care, confined to bed or chair more than 50% of waking hours, and grade 4; Completely disabled [21]. The eligibility criteria for the patients were as follows: age, 20 years or older; an appropriate medical status for cytoreductive surgery; advanced-stage epithelial ovarian, fallopian tube, and peritoneal cancer (FIGO stage III or IV); and FDG PET/CT performed less than 90 days before surgery. To exact measurement of metabolic parameters before surgery, patients who received neoadjuvant chemotherapy were excluded. Fifty-one patients who underwent primary cytoreductive surgery between 2011 and 2015 met the eligibility criteria for the current study. Suboptimal cytoreduction was defined as > 1-cm residual disease, as classified by the Gynecologic Oncology Group [22]. After primary debulking surgery, all patients received adjuvant at least 6 cycles of chemotherapy with paclitaxel and carboplatin.

The clinical and pathological parameters were reviewed and retrieved, including age, FIGO stage, histology, tumor grade, preoperative CA 125 level, ascites, ECOG PS, surgical time, surgical procedures, and postoperative complications.

### Calculation of the peritoneal cancer index

The PCI was used to quantitatively assess cancer distribution in the peritoneum. It is based on calculation of the lesion size in each of the 13 abdominopelvic regions (numbered from 0 to 12 in a clockwise direction). A lesion size score of 0 indicates no visible tumor burden in the peritoneum. Lesion size scores of 1, 2, and 3 describe maximal tumor diameters of up to 0.5, 5.0, and >  5 cm, respectively. After summarizing the lesion size scores from all regions, the PCI can be calculated for each patient. The highest PCI that could be reached was 39 [23]. The PCIs in our study were calculated using surgical and histopathological reports.

### FDG PET/CT image acquisition

All patients fasted for at least 6 h, and their blood glucose levels were checked before administration of FDG. Patients with blood glucose levels higher than 150 mg/dL were rescheduled for examination, and treatment was administered to maintain a blood glucose concentration of < 150 mg/dL in all participants. Patients received intravenous injections of approximately 8.1 MBq of FDG per kg of body weight and were advised to rest for 1 h before acquisition of FDG PET/CT images. FDG PET/CT scans were performed using Discovery 600 and 690 (GE Healthcare, Milwaukee, WI, USA). Before the PET scan, for attenuation correction, a low-dose CT scan was obtained without contrast enhancement from the skull base to the thigh when the patient was supine and breathing quietly. PET scans were also obtained from the skull base to the thigh at 2.5 min per bed position. PET images were reconstructed with a 128 × 128 matrix and an ordered-subset expectation maximum iterative reconstruction algorithm.

### Image analysis

The PET/CT images were interpreted by two experienced nuclear medicine physicians, and a final consensus was achieved for all patients. A positive finding was defined as any focus with increased FDG uptake in comparison with the surrounding normal tissue. Foci attributable to normal physiology or benign variants, such as muscular exercise or an infectious pulmonary infiltration, were excluded from the analysis. The primary tumors underwent semiquantitative and volumetric analyses using the volume viewer software on a GE Advantage Workstation 4.6 (GE Healthcare). The SUVmax was obtained using the following formula: SUVmax = maximum activity in the region of interest (mBq/g)/(injected dose [mBq]/body weight [g]). Automatic measurements were used to delineate the volume of interest using an isocontour threshold method based on the SUV.

**Fig. 3:**
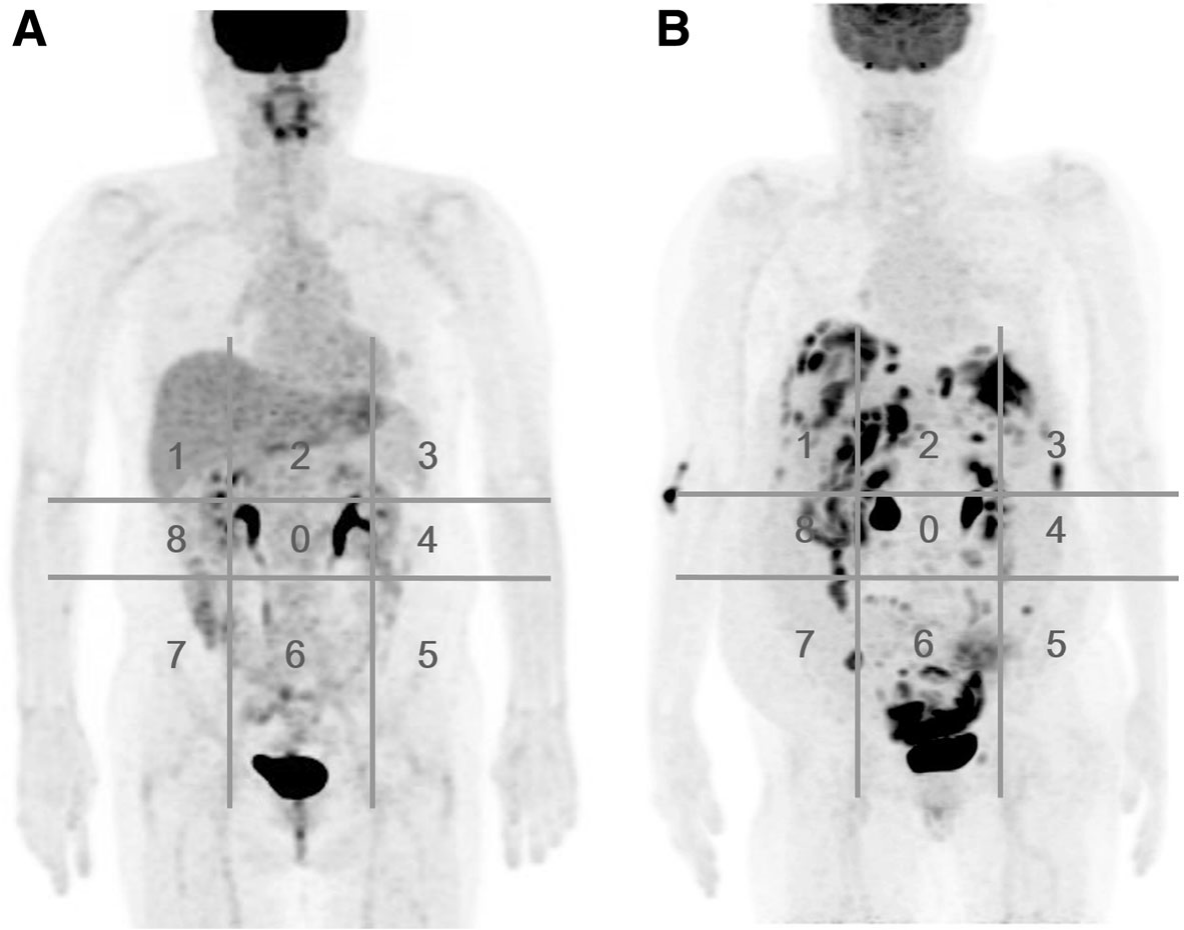
Representative F-18 FDG PET/CT images. F-18 FDG PET/CT images were analyzed according to 9 regions covering the whole peritoneal cavity including pelvis. Each quadrant was considered as a possible implant site and was evaluated separately. The regions were numbered as follows; 0, central; 1, right upper; 2, epigastrium; 3, left upper; 4, left flank; 5, lower left; 6, pelvis; 7, right lower; and 8, right flank. **a** F-18 FDG PET/CT images represents no evidence of peritoneal carcinomatosis in patients with ovary cancer. **b** F-18 FDG PET/CT shows ovary cancer patients with peritoneal carcinomatosis

To allow evaluation of peritoneal carcinomatosis, we used the internationally recognized PCI proposed by Sugarbaker [22] with a subtle modification, considering three anatomical regions: upper region (areas 1–3), middle region (areas 8–0-4), and lower region (areas 5–7) (Fig. 3). In addition, we included three sites of possible lymph node involvement, i.e., the pelvic, paraaortic, and extra-abdominal areas. SUVmax values of the nine areas and three lymph node sites were measured in each patient. The metabolic parameters of PET/CT were defined as follows: PET 0 = central, PET 1 = right upper, PET 2 = epigastrium, PET 3 = left upper, PET 4 = left flank, PET 5 = left lower, PET 6 = pelvis, PET 7 = right lower, and PET 8 = right flank. WB1SUVmax was defined as the sum of the SUVmax values for the nine abdominal regions, and WB2SUVmax was the sum of the SUVmax values for the three lymph node regions (pelvis, paraaortic, and extra-abdominal) and the WB1SUVmax.

### Clinical follow-up

Clinical follow-up examinations were performed every 3 months until 2 years, every 6 months after 2 years and up to 5 years, and annually thereafter. Failure was defined as biopsy-proven recurrence or documentation of disease progression on serial imaging studies. OS and DFS were chosen as clinical endpoints. OS was calculated from the date of diagnosis of disease to the date of death or last follow-up. DFS was calculated as the time interval from the date of diagnosis to the date of the first clinical or imaging findings that suggested disease recurrence.

### Statistical analysis

ROC curves were generated to determine the optimal cutoffs for continuous variables. Associations between the criteria and cytoreductive outcomes were tested using the Wilcoxon rank-sum test for continuous variables and Fisher’s exact test for categorical variables. Generalized estimating equations were used to account for differences between the two institution-clusters, assuming an independent covariance structure. The AUC was used as a measure of predictive accuracy. The clinical and radiologic criteria found to be significant in univariate analysis were then each assigned a “predictive score” according to their univariate ORs. The total predictive score of all patients in the cohort was subsequently calculated using each patient’s radiologic and clinical characteristics, and the suboptimal cytoreduction rate corresponding to each total score was determined. Survival curves of prognostic factors were estimated using the Kaplan-Meier method and the differences between subgroups were compared by a log-rank test.

All statistical tests were two-sided, and a *p* value of < 0.05 was considered significant. As the multivariate model was considered exploratory, no formal adjustment for multiple comparisons was made. Statistical analysis was performed using SPSS software v22.0 (SPSS, Chicago, Illinois, USA) and Medcalc version 15.4 (Medcalc Software, Ostend, Belgium).

## Abbreviations

AUC: Area under the curve
DFS: Disease-free survival
ECOG PS: Eastern cooperative oncology group performance status
FDG PET/CT: F-18 fluorodeoxyglucose positron emission tomography/computed tomography
FIGO: International Federation of Gynecology and Obstetrics
MTV: Metabolic tumor volume
OR: Odds ratio
OS: Overall survival
PCI: Peritoneal cancer index
ROC: Receiver operating characteristic
SUVmax: Maximum standardized uptake
